# Targeting FASN in Breast Cancer and the Discovery of Promising Inhibitors from Natural Products Derived from Traditional Chinese Medicine

**DOI:** 10.1155/2014/232946

**Published:** 2014-03-13

**Authors:** Chien-shan Cheng, Zhiyu Wang, Jianping Chen

**Affiliations:** School of Chinese Medicine, The University of Hong Kong, Pokfulam, Hong Kong

## Abstract

Molecular targeted therapy has been developed for cancer chemoprevention and treatment. Cancer cells process a fundamental change in its bioenergetic metabolism from normal cells on an altered lipid metabolism, also known as the *de novo* fatty acid synthesis, for sustaining their high proliferation rates. Fatty acid synthesis is now associated with clinically aggressive tumor behavior and tumor cell growth and has become a novel target pathway for chemotherapy development. Although the underlying mechanisms of the altered *de novo* fatty acid synthesis still remains unclear, recent progress has shown that by targeting Fatty acid synthase (FASN), a key enzyme that catalyzes the synthesis of endogenous long chain fatty acid could be a critical target for drug discovery. However, relatively few FASN inhibitors have been discovered. With the long history of clinical practices and numerous histological case study reports, traditional Chinese medicine enjoys an important role in seeking bioactive anticancer natural compounds. Herein, we will give an overall picture of the current progress of molecular targeted therapy in cancer fatty acid synthesis, describe the advances in the research on natural products-derived FASN inhibitors and their potential for enhancing our understanding of fatty acids in tumor biology, and may provide new therapeutic moieties for breast cancer patient care.

## 1. Introduction

### 1.1. Epidemiology of Breast Cancer

Breast cancer is the most prevalent cancer and the second leading cause of mortality in women worldwide [1]. According to the World Cancer Report [2], breast cancer comprises 22.9% of all cancers in women with an estimated 1.4 million new cases annually, resulting in more than 458,000 deaths in 2008 [2]. It was estimated that more than 1.6 million new cases of breast cancer emerged worldwide in 2010 [1, 3]. Since most breast cancers primarily affect women aged 50 and older, there is a clear relationship between menopause and breast cancer incidence [3–5]. During and after the onset of menopause, changes in hormonal status and in other metabolic processes contribute to the formation of a favorable microenvironment for the development of breast cancer [5, 6]. Several breast cancer treatment options have been considered to be conventional strategies in the last century, namely, mastectomy, chemotherapy, and radiotherapy, or a combination of the three [3]. With the rapid development of molecular medicines, novel therapeutic approaches, such as hormonal therapy and molecular targeted therapy, have been proposed to improve clinical outcome; however, the outcome of such approaches is still not ideal [3, 5, 7].

### 1.2. Role of TCM in Drug Development

Over the past few years, the development of complementary and alternative medicine (CAM) has drawn great attention in cancer research [8–12]. According to Newman and Cragg (2012), of the 175 small molecules approved worldwide as antitumor drugs during the period from 1981 to 2010, among the 74.8% nonsynthetic drugs, 85, or 48.6%, of them are either natural products or directly derived from natural products [13]. During the period from 2002 to 2010, of the 110 new drugs approved for cancer treatment, 37 of them originated from natural products [13]. TCM has thousands of years of experimentation on human beings. It has recently proved to be an important source for herb selection for drug discovery. In addition, TCM formulas provide promising sources for a more effective and less toxic treatment option for cancer [8, 10, 14–16]. The increasing interest and progress in seeking natural products has not only provided a chemical understanding of herbal drugs and their antitumor function but also contributed to the chemical bank for drug discovery [8, 13].

### 1.3. Obesity and Breast Cancer

In recent years, there has been increasing interest in the relationship between obesity and cancer. Obesity has been identified as the second leading cancer risk factor, after tobacco, especially for breast cancer [17, 18]. Statistics on breast cancer incidence and body fat indicate that those who reside in certain geographical areas, characterized by consumption of a more energy-dense diet, are at higher risk for breast cancer occurrence [1, 3, 19]. In addition, there is also a relation between body size and breast cancer risk. Women with a BMI of 30 versus 20.0–24.9 kg/m^2^ have an 82% increased risk for advanced cancer and a 22% increased risk for localized cancer [4, 19–21]. *De novo* lipogenesis, also known as *de novo* fatty acid synthesis, is the metabolic pathway synthesizing fatty acids, one of the essential substances for mammals, from nonlipid precursors, which is then incorporated with dietary and adipose tissue-derived fatty acid to produce triglyceride for energy storage that occurs in both liver and adipose tissue [22–24]. Adipose tissue expands to accommodate the excessive endogenous and exogenous lipid synthesized [22, 24]. *De novo* fatty acid synthesis is a highly regulated metabolic process and any of its dysregulation may result in various diseases [22, 25, 26]. An increase in *de novo* fatty acid synthesis plays an important role in increased fat mass and is an important contributor to pathological obesity [4, 22]. Obesity may affect not only the occurrence of breast cancer but also the prognosis of it, due to its influence on multiple pathways, such as hormonal influence, associated adverse disease features, and other yet unknown mechanisms [17, 21, 27–29]. There may be an interrelationship between obesity, altered lipid metabolism, and breast cancer risk, disease occurrence, and progression [4, 17, 21]. Therefore, the prevention of obesity and therapeutic approach of treating lipid metabolism disorders have risen as new approaches towards the prevention of breast cancer.

## 2. FASN as a Target for Cancer Therapy

### 2.1. Abnormal Fatty Acid Synthesis in Breast Cancer

**Figure 1:**
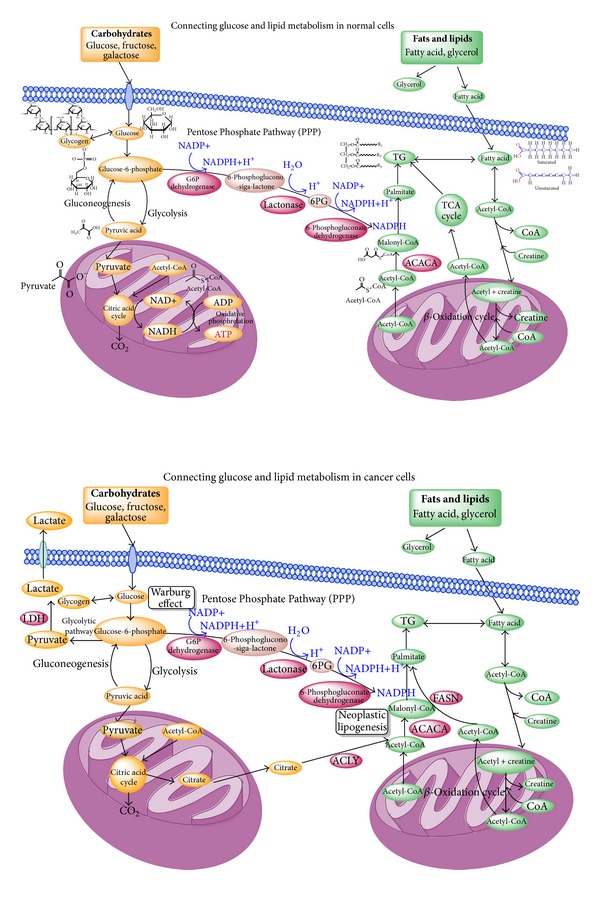
Connecting glucose and lipid metabolism in normal cells. The high rate of *de novo* synthesis generates essential lipids that are critical for the formation of cell membrane and produces extra energy for cancer proliferation and progression. In contrast to fatty acid synthesis being downregulated in most normal human tissues, precancerous lesion and cancer cells upregulate fatty acid synthesis, resulting in the high expression of FASN independent of dietary fat. Fatty acid synthesis also appears to be independent of hormonal regulation in many cancers including breast cancer. The inhibition of fatty acid synthesis results in the accumulation of the substrate Malonyl-CoA, which can create cytotoxicity and induce apoptosis. [30, 31].

Fatty acid is one of the essential substances for mammals [33]. It originates both externally (from a dietary source) and internally (synthesized by the *de novo* fatty acid synthesis) [34–37]. The internal sources of fatty acid, its related enzymes, and its relation to breast cancer tumorigenesis are the focus of discussions, rather than its external source [38, 39]. Cancer cells differ from normal cells in several ways [39]. There is a fundamental difference in the bioenergetic metabolism of aggressive cancer cells compared to normal cells [39]. Cancer cells, in order to sustain their high proliferation rates, rely not only on glycolysis, which is known as the “Warburg Effect”, but also on an altered lipid metabolism [15, 39]. Whereas normal cells prefer to utilize exogenous fatty acid from dietary sources cancer cells utilize a high rate of *de novo* fatty acid synthesis independent of dietary fat and independent, it appears, of hormonal regulation in many cancers, such as colon cancer and breast cancer [17, 40, 41]. The high rate of *de novo* synthesis generates essential lipids that are critical for the formation of cell membrane and produces extra energy for cancer proliferation and progression [22, 25, 42]. In contrast to fatty acid synthesis being downregulated in most normal human tissues, precancerous lesion and cancer cells upregulate fatty acid synthesis resulting in the high expression of FASN independent of dietary fat and independent of hormonal regulation in breast cancer (Figure 1) [17, 43].

### 2.2. Highly Expressed FASN in Breast Cancer

Research has shown that FASN is highly expressed in the hepatocytes and adipocytes of obese patients and in various kinds of cancer cells [17, 44, 45]. Therefore, targeting FASN has come to the attention of many in the drug industry due to the discovery that the inhibition of *de novo* synthesis of fatty acid can be used to treat cancer patients [17, 46]. Since the 1980s, researchers have identified FASN in various cancers including breast cancer, colon cancer, prostate cancer, ovarian cancer, and endometrium cancer [33, 42, 47]. The aforementioned cancers have shown that the FASN expression in these cancerous cells is significantly higher than that in the surrounding normal tissues [48]. Research has also shown that, through the inhibition of FASN activity or the reduction of its expression, cancer growth could be effectively reduced or apoptosis could be induced [23, 49–51]. However, the underlying mechanism is still under investigation. Some researchers claim that the inhibition of FASN will reduce the formation of essential fatty acid that is needed for the growth of cell membrane and for energy supply, which may be the cause of cancer cell apoptosis [34, 48, 52–54]. Other research findings suspect that the rise in Malonyl-CoA concentration is the trigger for cell apoptosis [53, 55]. Another research has shown that, after FASN inhibition, the growth of the cancer cell remains at G0 phase, showing that fatty acid synthesis is related to cancer cell cycle [46, 56, 57]. In short, the inhibition of FASN can effectively and widely inhibit the DNA replication of cancer cells and delay the S phase in the cell cycle, demonstrating that the pathway of fatty acid synthesis and DNA synthesis activity is related to the growth of cancer cells [58, 59].

In breast cancer, FASN is found to be highly expressed in various breast cancer cell lines, including hormone-independent SKBR3 and hormone-dependent MCF-7 and ZR-75-1 cells [45, 48, 60]. Among hormone-independent and hormone-dependent cell lines, hormone-independent breast cancer cells SKBR3 expressed higher (~2.5-fold) levels of FASN compared to hormone-dependent breast cancer cells [36, 61]. Aside from FASN expression differences between hormone-dependent and hormone-independent cell lines, FASN levels also increased with each successive tumor stage [36]. Research has demonstrated that the inhibition of fatty acid synthesis induces apoptosis and creates cytotoxicity which is likely to trigger cell death as a result of the accumulation of the substrate Malonyl-CoA (Figure 1) [30, 31]. However, scientists have not yet come to the agreement on the reason why and the specific mechanism by which the inhibition of fatty acid kills cancer cells [55, 62–64].

### 2.3. The Structure of FASN

Fatty acid synthase (FASN) is the key enzyme for *de novo* fatty acid synthesis and it catalyzes Acetyl-CoA and Malonyl-CoA to form Palmitate and 16-carbon long fatty acid [31, 65]. FASN has seven functional domains, namely, acyl carrier protein (ACP), Malonyl/acetyltransferase (MAT), ketoacyl synthase (KS), ketoacyl reductase (KR), dehydrase (DH), enoyl reductase (ER), and thioesterase (TE) [33, 35, 66]. It can be divided into two subtypes, types II and I. FASN in bacteria and plants is type II FASN with the seven functional domains independent of one another, which form a multicatalytic function system. On the other hand, FASN in humans and other mammals belongs to type I and is encoded by the FASN gene and composed of two identical 272 kDa multifunctional polypeptides, where the seven functional domains form a single bond [33, 37, 49, 66]. A person with sufficient nutrition who consumes a balanced diet undergoes little *de novo* synthesis of fatty acid. In such case, FASN expression is controlled at a low level because dietary lipid will provide the fatty acid needed for normal body functions [25, 42, 47, 54, 58, 67]. FASN has three main functions: energy storage, synthesis of fat, and lactation [33, 47, 58]. In highly lipogenic tissues, *de novo* fatty acid synthesis produces fat from excess carbon or energy intake and stores it as triglycerides [22, 68]. In a lactating female, FASN produces medium chain fatty acids along with thioesterase-II and this enables the baby to easily digest the milk [34–36]. Under normal conditions, FASN is regulated by one's diet and hormones. All carbohydrate intake, thyroid hormone, insulin, and glucocorticoid can up-regulate fatty acid synthesis, while unsaturated fatty acid, cAMP, and glucagon can down-regulate FASN and fatty acid synthesis [4, 42, 45, 69, 70].

### 2.4. The Metabolism and Expression of FASN

**Figure 2:**
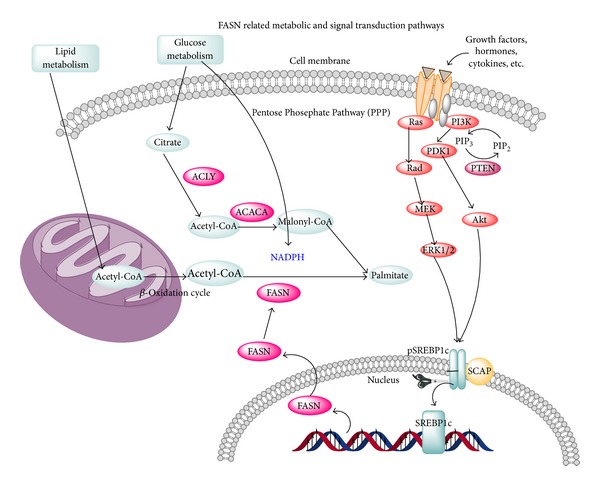
FASN related metabolic and signal transduction pathways. *De novo* fatty acid synthesis is an important hallmark of cancer cells, differentiating it from normal cells. Palmitate is the primary synthesized 16-carbon fatty acid, from which other lipids are also formed. FASN is the key enzyme for the synthesis of palmitate and it affects the amount of palmitate formed. The overexpression of FASN allows for the *de novo* synthesis of essential lipids for the formation of cell membrane and for the production of extra energy via beta-oxidation and lipid modification of proteins. The binding of the growth factor and the growth factor receptor results in the activation of their downstream PI3k-Akt and ERK1 (and ERK2) signal transduction pathway. The FASN expression is regulated by several growth factors, including epidermal growth factor receptor (EGFR), HER2, steroid hormone, and steroid hormone receptors (such as ER, PR, and AR). However, there is complicated regulation of FASN expression by these growth factors. FASN expression and growth factor-dependent signaling are coregulating.

In the last decade, numerous studies have focused on the metabolic alternation of adaptation of cancer cells [71]. Cancer cells possess the feature of high proliferation differentiation [72]. Cells constantly divide, grow, and absorb external nutrition, while in the meantime self-synthesizing essential material for growth [72, 73]. *De novo* fatty acid synthesis is an important hallmark of cancer cells setting it apart from normal cells [42]. Lipids are the main component for the composition of cell membrane and are essential for cell division [27, 74, 75]. Palmitate is the primary synthesized 16-carbon fatty acid, from which other forms of lipids are also formed [76]. FASN is the key enzyme for the synthesis of palmitate and affects the amount of palmitate formed (Figure 2) [18, 35, 42, 44]. FASN is expressed in most of cancer cells, for example, breast cancer cells, and has a low expression in normal cells [33, 66]. With *de novo* fatty acid synthesis, tumor cells endogenously synthesize extra fatty acid allowing them to sustain higher proliferation rates and faster growth [25, 77]. Therefore, targeting fatty acid synthase can be an important strategy for cancer prevention and treatment.

### 2.5. Signaling Pathway and Regulation of FASN

**Figure 3:**
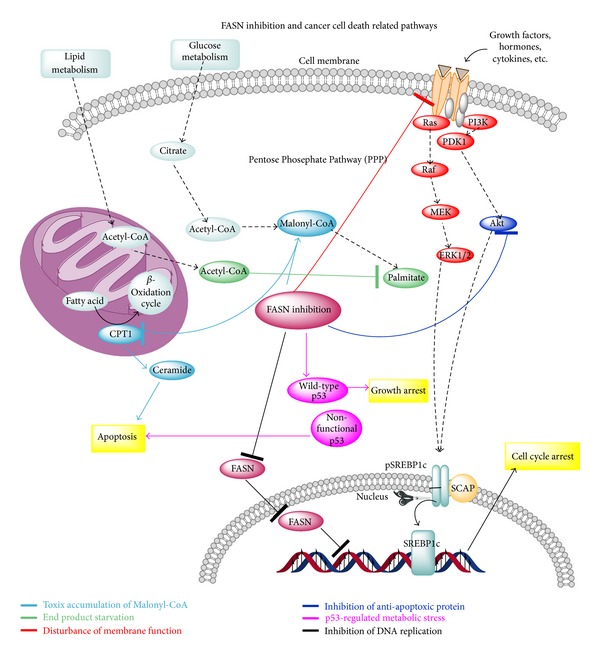
FASN inhibition and cancer cell death related pathways. Inhibiting FASN by either drug or by gene silencing can inhibit HER2 expression; meanwhile, HER2 overexpression leads to an increased expression of FASN, indicating that there is a bidirectional regulation mechanism between FASN and HER2. FASN expression can be not only regulated by SPEBT-1c but also controlled by other transcription factors, such as p53 family proteins and the lipogenesis-related nuclear protein, SPOT14, which is overexpressed in breast cancer. The steroid hormones binding with steroid hormone receptors can also activate similar pathways. These two pathways stimulate FASN expression by the gene modulation and/or the nuclear maturation of the sterol regulatory element-binding protein 1c (SREBP1C), which is a transcription factor that activates FASN by binding to its promoter region, which contains sterol regulatory elements [32]. Currently, there are several pathways that contribute to the initiation of FASN cytotoxicity. One is where FASN inhibition initiates a more effective apoptosis in cells with nonfunctioning p53 protein compared to those cells with functioning p53 protein, which occurs concurrently with cytostatic response. One is where HER2 overexpressed cells are also linked to FASN induced cytotoxicity. In addition, there are studies on ovarian cancer cell lines showing that FASN activity modulates Akt activation, and, at the same time, Akt activation regulates FASN expression, suggesting that the Akt activation protects cells against FASN inhibitor induced cell death.

The overexpression of FASN allows for the *de novo* synthesis of essential lipids for the formation of cell membrane and for the production of extra energy via beta-oxidation and lipid modification of proteins [55, 71, 78]. Although the mechanisms for tumor related FASN overexpression are still unclear, two main mechanisms have been proposed. The first one is the growth factor, which binds to growth factor receptor, activating their downstream PI3k-Akt and ERK1 (and ERK2) signal transduction pathway (Figure 2) [34, 42, 45, 47, 68]. The FASN expression is regulated by several growth factors, including epidermal growth factor receptor (EGFR), HER2, steroid hormone, and steroid hormone receptors (such as ER, PR, and AR) [27, 47, 55, 62, 77]. However, the regulation of FASN expression by these growth factors is rather complicated. FASN expression and growth factor-dependent signaling can be coregulated [42, 49, 50]. For example, through downstream PI3K signaling, HER2 regulates FASN expression; meanwhile, reports also demonstrate that HER2 expression is regulated by FASN expression [45, 57, 64]. HER2 is an overexpressed gene in cancer cells [7, 38, 62, 77]. HER2 is the Tyrosine kinase receptor, which belongs to erbB family [23, 62, 63]. It is similar to the epithelial growth factor receptor and is also involved with regulative cell growth and division [62]. HER2 is expressed in various types of cancer cells [55, 62]. Through overexpressing HER2, cancer cells can produce drug tolerance toward chemo drugs and are related to poor prognosis in cancer patients. Recent studies have shown that FASN and HER2 are both overexpressed in certain types of cancer cells [46, 55, 62, 77]. Inhibiting FASN by either drug or gene silencing can inhibit HER2 expression; meanwhile, HER2 overexpression leads to an increase expression of FASN, indicating that there is a bidirectional regulation mechanism between FASN and HER2 (Figure 3) [25, 34–36, 79].

Recent studies have shown that FASN expression can be not only regulated by SPEBT-1c but also controlled by other transcription factors, such as p53 family proteins and the lipogenesis-related nuclear protein, SPOT14, which is overexpressed in breast cancer [25, 47, 79, 80]. The second mechanism is one whereby steroid hormones binding with steroid hormone receptors activate similar pathways [35]. These two pathways stimulate FASN expression by the gene modulation and/or the nuclear maturation of the sterol regulatory element-binding protein 1c (SREBP1C), which is a transcription factor that activates FASN by binding to its promoter region, which contains sterol regulatory elements [32, 35, 70]. Currently, there are several pathways that contribute to the initiation of FASN cytotoxicity. One is where FASN inhibition initiates a more effective apoptosis in cells with nonfunctioning p53 protein compared to those cells with functioning p53 protein, which occurs concurrently with cytostatic response [24, 32, 59, 62]. One is where HER2 overexpressed cells are also linked to FASN induced cytotoxicity. In addition, there are studies on ovarian cancer cell lines showing that FASN activity modulates Akt activation, and, at the same time, Akt activation regulates FASN expression, suggesting that the Akt activation protects cells against FASN inhibitor induced cell death (Figure 3) [35, 62–64].

### 2.6. Therapeutic Implication Progress of FASN in Cancer Treatment, Diagnosis, and Prevention

There are two unique characteristics of FASN that made it suitable for being an antitumor target: its tissue distribution and its enzymatic activities. FASN is highly expressed in breast cancer but not in nonlactating normal breast tissue [35, 47, 52, 81]. From the enzymatic activities aspect, it catalyzes the terminal step of *de novo* fatty acid synthesis, not affecting other important components of the lipid metabolism [34, 42, 68]. In addition to its multienzymatic functions characteristics, it contains seven catalytic domains that can be targeted, as mentioned before. Thus, using FASN as a target will influence the proliferating compartment of the breast while the nonproliferating compartment will remain unaffected [23, 35, 47].

## 3. Natural Product-Derived FASN Inhibitors

### 3.1. Single Compound

#### 3.1.1. Single Domain

*(1) KS Domain*. Currently, there is limited amount of research on FASN inhibitors. Cerulenin is the first FASN inhibitor identified and is a natural metabolic product used in antibiotics [33, 34, 61, 77]. It is a noncompetitive inhibitor with FASN and covalently binds with the hydroxyl group on serine at the end of the KS domain, forming hydroxyl-beta-lactam, irreversibly inhibiting *de novo* fatty acid synthesis [63, 80]. Cerulenin mainly inhibits the synthesis of long chain fatty acids and only inhibits the growth of cancer cells while the normal cells remain unaffected [63, 80, 82]. However, the usage of Cerulenin is limited because of its unstable structure and high toxicity levels [80, 82, 83].

*(2) TE Domain*. Other FASN inhibitors that can potentially be used as cancer treatment drugs have been identified, such as Orlistat (1-(3-hexyl-4-oxooxetan-2-yl) tridecan-2-yl 2-formamido-4-methylpentanoate), a drug designed to treat obesity and marketed as a prescription in most countries [46]. Recent research has shown that Orlistat may also act on the TE domain of the FASN [46, 48]. Currently, Orlistat is the only FASN inhibitor under clinical usage. Other derivatives from Orlistat, such as the beta-lactam derivatives of Orlistat identified, have shown good inhibitory effect on FASN activity [46, 48, 49]. Therefore, the development of Orlistat and its derivatives will be an important approach for future research on FASN inhibitors.

*(3) KR Domain*. Epigallocatechin-3-gallate (EGCG), a natural component of green tea, is one of the several natural plant-derived polyphenols identified to have an FASN inhibitory effect higher than that of C75 [83–85]. EGCG acts on the KR domain of the FASN and is a high micromolar time-dependent inhibitor [83, 86, 87]. EGCG has a prominent inhibitory effect because it not only acts on the FASN but also could inhibit HIF-1-alpha expression by blocking PI3K/Akt signaling pathway [87]. Recently, EGCG has been developed as a tumor growth inhibitor and is used in breast cancer xenograft models. Even so, there are limitations for EGCG as an FASN inhibitor, and its FASN inhibition effect can still be improved upon [77, 83, 86, 87]. Prior experiments have shown that, after the strong acid and heating treatment of EGCG, FASN inhibition will be greatly increased, and this raises the possibility that the increased effect is due to the change in the chemical composition of catechin [83, 87]. However, the follow-up experiment showed that EGCG becomes unstable after strong acid and heat treatment [83, 86, 87]. The initial product is unstable as well, which makes it difficult to investigate its molecular composition and properties.

Other FASN inhibitors include urea-derived compounds such as GSK837149A and can specifically inhibit the KR domain of FASN [88].

#### 3.1.2. Multiple Domains

In 2000, Lotfus and his fellow colleagues reported a small molecule, a structurally modified form of Cerulenin to be a novel FASN inhibitor, the C75 [89]. It is a derivative of 3-carboxy-4-alkyl-2-methylenebutyrolactones that overcomes the toxicity of Cerulenin and performs a better specific inhibitory effect than Cerulenin [36, 90]. Research has shown that the inhibitory effect of C75 on FASN differs from that of Cerulenin [36, 49, 77, 90]. C75 inhibits FASN with a noncovalently reversible inhibition to the KS, ER, and ES domain of FASN [77, 90]. The mechanism, however, by which C75 inhibits cancer cell growth, is still unclear [80, 90]. In 2000, Kuhajda et al. demonstrated that C75 inhibits fatty acid synthesis inducing cancer cell apoptosis; however, more in-depth research is still needed [36, 81]. Although, compared to other FASN inhibitors, much research has focused on C75, its clinical usage is limited due to its unstable nature [77].

#### 3.1.3. Without Inhibitory Target Domain Studies

Icaritin (ICA), a prenylflavonoid derivative from *Epimedium* Genus, can effectively induce SKBr3, ER negative breast cancer cell line, and cell apoptosis and suppress cell growth in a concentration-dependent manner [91]. Through the FASN/HER2 pathway, ICA is unable to upregulate PEA3 and downregulate HER2 [91]. This suggests that ICA can be a potent FASN inhibitor and a potential anticancer drug for future development.

Amentoflavone, a natural biflavonoid isolated from *S. tamariscina* and abundantly found in the family *Selaginellaceae*, can suppress FASN expression at the protein and mRNA levels [62, 92]. *S. tamariscina* has a long history of being used for the treatment of advanced cancer in TCM [62, 78], as well as being used as an antioxidant, vasorelaxant, and anti-HIV and antiangiogenesis agent [62, 78, 93, 94]. In addition, despite amentoflavone's FASN-inhibitory effect, which inhibits the translocation of SREBP-1 in SKBR3 cells, it can suppress HER2 activation and modulate Akt, mTOR, and JNK phosphorylation [32, 62, 94]. These actions induce cell death and increase the potential for using amentoflavone as a chemotherapeutic drug in the prevention or treatment of HER2-positive breast cancers [78].

### 3.2. Extract

#### 3.2.1. With Inhibitory Target Domain Studies

Many researchers have found that EGCG extracted from grape seeds has a better inhibiting capacity compared to the effects of Monomeric catechin and epicatechin on FASN inhibition explored in previous research [95]. Therefore, the separation of an active compound of grape seed extract will be meaningful for future studies in terms of its use as a novel FASN inhibitor, but there are many steps of extraction that will need to be further improved [83, 87, 95]. Thus, future investigation and study are needed to confirm the molecular mechanism of the increased FASN inhibition effect of EGCG as well as search for the conditions necessary to stabilize the formation of a high-activity and stable FASN inhibitor.

Coix seed extract from Semen Coicis is an oily substance, and its main active ingredient is a triglyceride containing four fatty acids [96]. It is the first drug derived from TCM to go into clinical trials in the USA and has been proved to be an effective and safe TCM practice by preclinical antitumor studies and pharmacokinetics and safety studies [97]. Yu et al. studied the Coix seed extract's FASN inhibitory effect [96, 97]. Their experiment in vitro demonstrated that Coix seed extract, by inhibiting the KR and ER domain of FASN, could significantly inhibit FASN activity; in addition, their experiments in vivo also observed a significant FASN inhibitory effect in the liver produced by the Coix seed extract, which was accomplished by elevating LPL and HL activity in the plasma as well as affecting the G-6-PD activity [96, 97]. Thereby, Coix seed extract inhibits fatty acid synthesis and reduces formation of energy supplying tumor cell growth. Therefore, the antitumor effects of Coix seed extract are likely to be related to the inhibition of FASN activity [96, 97]. The study supports that FASN is a novel target for cancer therapy. Other studies of Coix seed extract show that it can inhibit tumor cell mitosis at G2/M phases, induce tumor cell apoptosis, inhibit tumor angiogenesis, and also upregulate FASN/Apo-1 gene expression and downregulate Bcl-2 gene expression of tumor cells. In addition, it can prevent anticancer drug resistance in tumor cells [96, 97]. The present study supports FASN as a novel target for cancer therapy and provides a theoretical foundation for the clinical application of Coix seed extract in cancer therapy. Continued studies are required for the future clinical uses of Coix seed extract as an FASN inhibiting anticancer drug.

A research by Wang et al. indicates that extracts of *Fallopia multiflora* can both reversibly and irreversibly inhibit FASN and exhibit a better inhibitory effect than both Cerulenin and C75 [98]. Different than that of C75, which can only inhibit the KS domain of FASN, *Fallopia multiflora* extract can both inhibit the KR and the MT domain [98, 99]. However, due to the complexity of the herbal extract, the specific analog that exhibits the inhibitory effect has not yet been identified [99]. Future research is required for a better understanding of the pharmacological activity and the inhibitory mechanism in order to develop better FASN inhibitors in the future. The screening results may provide direction for future research on novel FASN inhibitors from these categories of herbal medicines or compounds with similar chemical structures.

### 3.3. Only with Basic Inhibitory Effect Screening

Allium vegetables, represented by Allium *fistulosum *L., have been employed for a long time in traditional medical practice to treat a variety of diseases [100, 101]. In 1998, a case-control epidemiological study carried out in France suggested that higher onion intake was a protective factor for breast cancer [100]. Recently, the anticarcinogenic potential of onion had been extensively studied by Wang et al. in 2013 [102]. The study demonstrated that onion extract can inhibit FASN activity and induce MDA-MB-231 cells [64, 102] apoptosis as well as reducing intercellular lipid accumulation of 3T3-L1 adipocytes [102]. This raised the potential of developing onion into a drug for the prevention and treatment of breast cancer as well as other obesity-related cancers [100–102].

*Nephelium lappaceum* L., commonly known as “rambutan”, is a tropical fruit of the *Sapindaceae* family whose antioxidant and antibacterial functions have been used for severe dysentery or as a carminative in dyspepsia [103, 104]. An article published in 2011 by Zhao et al. found that *Nephelium lappaceum* L. has an FASN inhibitory effect comparable to the commonly known natural FASN inhibitor EGCG [104]. This revealed the potential of *Nephelium lappaceum* L. for being a promising FASN inhibitor. In addition, isolated glycosides from *Nephelium lappaceum* L., the oleane-type triterpene oligoglycosides, may also be another type of FASN inhibitor which may be developed into a novel chemoprevention and chemotherapy drug in the future [103, 104].

There have been years of research on FASN inhibitors and their role in breast cancer therapy in western medicine and recently Chinese Medicine has developed a similar research, with a focus on the weight loss effect of the inhibition of FASN using herbs [105]. Tian et al. performed a screening of FASN inhibitor in 31 common herbal medicines and discovered a number of effective inhibitors in 2004 [105]. Among the 31 herbs, *Polygonum multiflorum *(*何首烏*) [106, 107], *Alpiniae officinarum* (*高良姜*), *Parasite scurrula* (*桑寄生*), to name a few, showed strong FASN inhibitory effect and weight loss effect in animal models [108]. Both *Alpiniae officinarum* and *Parasite scurrula* contain phenols such as flavone, increasing the potential for polyphenols to be an important active substance in terms of FASN inhibition [105, 108].

Recent research has screened potential Chinese herbal medicines for their inhibition effect on fatty acid synthase. Out of the 225 commonly prescribed herbal medications screened, 58 of them (25.78%) show strong (80–100%) inhibitory effect [109]. Among those 58 strong potential FASN inhibitors, some pure compounds were found including alkaloid, anthraquinone, flavonoid (e.g., dried leaf of *Apocynum venetum* L. (*羅麻布葉*), spine of *Gleditsia sinensis* (*皂角刺*), *Spatholobus suberectus* (*雞血藤*) [60], *Prunella vulgaris* (*夏枯草*), *Scutellaria baicalensis* Georgi (*黃芩*), hawthorn fruit, and pagodatree flower), phenols, kaempferol, tannins (e.g., *Melaphis chinensis* (Bell.) (*五倍子*), the root of *Sanguisorba officinalis* L. (*地榆*), *Rhuen palmatum* L. (*大黃*), and mature seed of *Litchi chinensis* Sonn (*荔枝核*)), triterpenes, gallic acid, ursolic acid, paeoniflorin, and saponins [105, 109, 110]. These herbs are characterized, according to Chinese medicine theory, into those which have the effects of clearing away heat and toxic materials (Table 1), enriching the blood and invigorating the circulation of blood (Table 2), promoting health while promoting meridian and blood (Table 3), regulating Qi Flow (Table 4), and reducing phlegm and dehumidification (Table 5) and other effects (Table 6) including removing blood stasis and promoting the subsidence of swelling, analgesic, warming spleen and stomach for dispelling cold, and cooling blood to stop bleeding by their functions. Linoleic acid [50], some poly-unsaturated acid [50, 100], polyphenols [108], and flavones [94], among others, have all shown some FASN inhibitory effects, with some displaying better inhibiting effects than C75 [109].

## 4. Future Perspective and Conclusion

The *de novo* fatty acid synthesis is a distinct feature of cancer cells, distinguishing it from normal cells [35]. The tumor cell's intrinsic properties and the tumor microenvironment result in the metabolic alternation and adaptation of cancer cells. This lipid metabolism alternation and the high rate of *de novo* fatty acid synthesis allow cancer cells to sustain higher proliferation rates and rapid growth. Therefore, targeting the key enzyme of the fatty acid synthesis, the FASN, can be an important strategy for anticancer drug development.

### 4.1. CAM as the Important Trend in Cancer Treatment and the Superiority of TCM in FASN Inhibitors Development

Complementary and alternative medicine (CAM) has been a recent source of drug discovery in cancer research. A wide range of prescription drugs used for cancer treatment are derived from medicinal plants and 74% of those drugs were based on “folk medicine” [111]. Among all CAM modalities, TCM has a long history of clinical practice and well-established theoretical approaches and also enjoys particular appreciation for its uses as an adjunct therapy to slow down the development of chemotherapy drug resistance and to relieve the unpleasant side effects of chemotherapy and radiotherapy [60]. However, although TCM is appreciated for its efficiency and minimal side effect, there is still insufficient understanding of its mechanism as well as inadequate evidence for verifying its effectiveness [9, 15, 111]. Although both the standard cancer therapy and TCM have been used for decades in China for improving both the clinical outcome in cancer treatment and the quality of life of the surviving patients, [11, 111] due to insufficient quality control for herbal products and the shortage of randomized controlled trials (RCT), whether herbal medicines can indeed treat cancer remains controversial [10–12, 15].

Statistics have shown that out of the 260,000 to 300, 000 plant species in the world, only around 5,000 of them have been studied for their possible medical applications [15]. With the long history of clinical based practice of Chinese herbal medicine and great number of histological document and case reports in cancer treatment, Chinese herbal medicine has a unique position in drug discovery for molecular targeting based therapeutic strategy [9–11, 15]. The research done on FASN inhibitors is receiving increasing amounts of attention from the media, since it was reported as a potential therapeutic target for both obesity and breast cancer. This has raised the question of whether there are any potent FASN inhibitors in Chinese herbal medicine or in any other natural product. The ongoing development of the FASN inhibitors screening strategy will provide a good platform for exploring and discovering inhibitors in natural products, which are more molecular target specific and contain lower toxicity levels. Chinese herbal medicine with its multiple active components is considered a promising source for the design of drugs that regulate multiple cellular signal pathways for cancer therapy. This leads us into a new era or anticancer drug development.

### 4.2. Future Potential of FASN Inhibitors in Cancer Treatment

As mentioned before, molecular targeted therapy has been attracting considerable attention in the research of cancer treatment. An ideal molecular target should be preferentially expressed or activated in malignant cells but not in normal cells, thus making FASN an attractive target for future research. With the progress of molecular and genetic technologies, a number of target inhibitors have been discovered either by synthesis or design. In FASN specific inhibition, C75 and EGCG are well known examples of inhibitors. However, their therapeutic efficacies are limited due to either their high toxic level or unstable nature. Therefore, seeking a more stable and potent drug as a target inhibitor will be an important future trend for research and development for oncologists.

In the future research for potential FASN inhibitors, there should be two main approaches: the first is to screen for individual herbal medicines and their active compounds and the other is to screen for traditional formulas or effective clinical experience-based formulas under the guidance of either TCM theory or modern pharmacology [9, 12]. On the basis of the theory of traditional Chinese medicine, the treatment of breast cancer with a focus on weight loss effect can follow two approaches: by smoothing liver and regulating Qi and by promoting blood circulation for removing blood stasis. Therefore, further research on herbal medicines and traditional formulas may be a promising method for the development of more potent anticancer drugs.

Over the past few decades, active compounds extracted from herbal medicines have received good responses clinically. The investigation of Chinese Herbal Medicine with weight loss effects, which may be related to inhibition of the fatty acid synthesis pathway, may be an advantageous approach for future research. It is worthwhile to investigate those herbal medicines commonly used in Traditional Prescription for antibreast cancer treatment as well, such as the root of *Sanguisorba officinalis* L. (*地榆*), *Spatholobus suberectus* (*雞血藤*), *Pericarpium granati* (*石榴皮*), the root of *Paeonia lactiflora* Pall. (*白芍*), *Paeonia vaitchii* Lynch (*赤芍*), and *Paeonia suffruticosa* Andr. (*牡丹皮*). Further studies on the inhibitory mechanisms and domains of these medicines, with their relatively good FASN inhibitory effect, will provide us with a much better understanding of cancer biology and equip us with the knowledge needed for better future implementation for future cancer chemotherapy and chemoprevention.

Currently, all of the basic screenings have only focused on the FASN inhibitory effect of one single herbal medicine. However, clinically, most of the Chinese herbal medications are prescribed as a combination of a number of herbal medicines, in the form of a decoction. There is limited research on the FASN inhibitory effect of TCM herbal decoctions. Some decoctions commonly prescribed to breast cancer patients have been found to be composed of several herbal medicines that have proven FASN inhibitory effects. As mentioned before, smoothing liver and regulating Qi and promoting blood circulation for removing blood stasis are two main ways to treat breast cancer under the guidance of TCM theory. Herein, we will give one example of TCM prescription with the effect of smoothing liver and regulating Qi (Tables 7-1); four examples (Tables 7-2,3,4,5) with the effect of promoting blood circulation for removing blood stasis; and one example with both of these two effects (Tables 7-6).

### 4.3. Conclusion

Unlike surgery, chemotherapy, and radiotherapy, which can have serious consequence on the human body, the practice and usage of Chinese herbal medicine in cancer therapy mobilize and regulate the body's functions, enhancing the body's ability to fight cancer. Therefore, Chinese herbal medicine may be more suitable for inoperable patients with advanced cancer, for those in intermittent periods of chemotherapy, or for patients in a postoperative recovery period. There has been an increase of attention on the research of Chinese herbal medicine in the prevention of cancer and inhibition of cancer metastasis. There is an impressive renaissance seeking for semisynthetic drugs or derivatives from natural compounds. Progress in this regard not only adds to the Chemical Bank but also leads to a better understanding of the chemical basis of Chinese herbal medicine for the treatment of cancer using drugs obtained from natural sources.

## Figures and Tables

**Table 1:** Herbs with clearing away heat and toxic material functions with strong FASN inhibitory effect. In TCM theory, noxious heat is one of the underlying pathologies of the occurrence and development of malignancy. Therefore, herbal medicine with detoxification effect plays an indispensable role in cancer prevention and treatment. With pharmacological studies and analysis of their chemical composition, the herbs anticancer mechanism mainly includes direct inhibition of tumor-induced apoptosis of tumor cells, regulation of immune function, anti-inflammatory, detoxification, fever, blocking, and anticarcinogenic mutations. These kinds of herbs are widely used in the clinical treatment of cancer.

*清熱* *解毒* Clearing away heat and toxic material	Chemical components	Pharmacological action
*Scutellaria barbata* (*半枝蓮*)	Alkaloid; Flavonoid glycoside; Phenol; Steroid; Stearic acid	Antitumor; antimutagenic effect; diuresis; eliminating phlegm; antivirus

The root of *Sanguisorba officinalis* L. (*地榆*)	triterpenoid saponin Tannins; Sanguisorbin; A,B,E Gallic acid; Ellagic acid	Antitumor; antibiosis; antiemetic; hematischesis

The root of *Sophora flavescens* Ait. (*苦參*)	Matrine; Oxymatrine; Anagyrine; Flavonoids	Antitumor, diuresis, bacteriostat

*Glechoma longituba* (Nabai) Kupr (*金錢草*)	Flavonoids, Quercetin; Choline; glycosides; Macrolide	Antitumor; detumescence; bacteriostat

*Prunella vulgaris *L. (*夏枯草*)	Flavonoids; Baicalin; Baicalein; Wogonin; Wogonoside	Antitumor, depressurization; diuresis; antibiosis

The root of *Scutellaria baicalensis *Georgi (*黃芩*)	Flavonoids; Baicalin; Baicalein; Wogonin	Antitumor; antibiosis; cholagogue; hepatoprotective effect

*Sophora japonica *L. (*槐花*)	Rutin; Triterpenoid Saponin; Flavonoids; Steroids; Tannins	Antitumor; antiulcer; protect blood vessels; hypolipidemic; anti-inflammation

**Table 2:** Herbs with promoting blood circulation for removing blood stasis functions with strong FASN inhibitory effect. Stagnation of Qi and poor blood circulation are recognized as the main pathologies of certain groups of malignancy. This group of herbal medications may not only induce apoptosis but also improve the antitumor mechanism of a body's blood circulation, which improves the local hypoxic state, as well as regulating and promoting immunity.

*活血* *化瘀* Promoting blood circulation for removing blood stasis	Chemical component	Pharmacological action
The fruit of *Crataegus pinnatifida *Bunge (*山楂*)	Crataegolic acid; Chlorogenic acid; Epicatechin; Epicatechol; Flavonoids	Blocking synthesis of nitrosamine

*Rosa chinensis *Jacq. (*月季花*)	Gallic acid	Antibreast cancer; antithyroid neoplasm

*Paeonia veitchii *Lynch (*赤芍*)	Paeoniflorin; Galloylpaeoniflorin; Paeonol; Lacioflorin; Catechin	Antitumor

*Paeonia suffruticosa *Andr. (*牡丹皮*)	Paeonol; Paeonoside; Paeonolide; Paeoniflorin; Gallic acid; Phytosterol; Alkaloid	Antitumor; immunoregulation; bacteriostat

*Spatholobus suberectus *Dunn (*雞血藤*)	Daidzein; Epicatechin; Protocatechuic acid; Brassicasterol; Stigmasterol; *β*-sitosterol; Auriculatin	Antitumor; bacteriostat

*Polygonum cuspidatum *Sieb. etc Zucc (*虎杖*)	Polydatin; Emodin; Physcion; Chrysophanol; Citreorsein; Anthraglycside; Resveratrol	Antitumor; immunoregulation; antibiosis; antivirus; elevation of white blood cell counts

*Herba lycopi* (*澤蘭*)	Essential oils; Flavonoid Glycosides; Saponins; Phenols; Tannins	Antitumor, immunoregulation

**Table 3:** Herbs with promoting health's function with strong FASN inhibitory effect. Under TCM theory, the weakness of a healthy atmosphere in the human body is one of the major underlying pathologies for malignance occurrences. It has been more widely accepted to palliative therapy without the resection of tumor. This group of herbs is commonly used in combination with chemotherapy and radiotherapy to improve the patients overall health and increase tolerance of chemotherapy.

*扶正* *培本* Promoting health	Chemical components	Pharmacological action
The root of *Paeonia lactiflora *Pall. (*白芍*)	Paeoniflorin; Paeonol; Paeonin; Triterpenoids; Essential oils; Tannins	Antitumor, improve immune function; composed; analgesia; relieve fever; anti-inflammation

*Cinnamomum cassia *Presl (*肉桂*)	Essential oils; Tannins, Mucoid substances; Resins	Antitumor, composed; analgesia

*Polygonum multiflorum *Thunb. (*何首烏*)	Emodin; Chrysophanol; Rhein; Resveratrol; Catechin; Epicatechin; Piceid; Gallic acid	Improve immune function; lowering serum cholesterol; treatment of atherosclerosis

*Fructus psoraleae* (*補骨脂*)	Essential oils; Saponins; Flavonoids; Astragalin; Bavachin; Monoterpenes; Resin; Stigmasterol;	Antitumor; bacteriostat

*Drynaria fortunei *(Kunze) J. Sm. (*骨碎補*)	Hesperidin; Naringin; Sting-masterol; Triterpenes	Antitumor; promote bone calcium absorption; lowering serum cholesterol; composed; analgesia;

*Epimedium brevicornum *Maxim. (*淫羊藿*)	Icariin; Phytosterol; Essential oils; Cetyl alcohol; Hentriacontane; Palmitic acid; Stearic acid; Linolenic acid; Bilobanol; Hyperin; Tannins	Antitumor; antibechic; enhance sexual function

*Cynomorium ongaricum *Rupr. (*鎖陽*)	Tannins; Anthocyanin; Triterpenoid saponin	Enhance immune function; demulcent

*Carica papaya *L. (*番木瓜*)	Papain; Carica; Rennet; Carotene	Inhibition of tumor growth

*Eclipta prostrata *L. (*墨旱蓮*)	Tannins; Essential oils; Saponins; Ecliptine	Hematischesis

**Table 4:** Herbs with regulating Qi flow's function with strong FASN inhibitory effect. The stagnation of Qi, one of the most important material bases in human body according to TCM theory, will result in a lot of different diseases including malignancy. Therefore, regulating the Qi flow is an important strategy in diseases treatment and in malignancy treatments; this group of herbs is usually used in combination with those stated in Tables 2 and 3.

*理氣* Regulating QI flow	Chemical components	Pharmacological action
*Illicium verum *Hook. f. (*八角茴香*)	Fatty oils; Anise oil; Resin; Quercetin; Kaempferol	Antitumor; elevation of white blood cells; bacteriostat; promote digestion

*Lindera strychnifolia *(Sieb. et Zucc.) F.-Vill (*烏藥*)	Essential oils; borneol; Furan sesquiterpene; Alkaloid; Neolinderalactone; Linderane	Antitumor; promote intestinal peristalsis; promote the secretion of digestive juice; antibiosis

*Rosa rugosa* Thunb. (*玫瑰花*)	Essential oils; Citronellol; Vernol; Phenylethyl acetate; Isoquercitrin; Tannins; Gallic acid	Antitumor; immunoregulation

*Fructus Amomi* (*砂仁*)	Bomyl acetate; Camphor; Limonene; nerolidol; Cineole; Flavonoids	Antitumor; bacteriostat

*Areca catechu* L. (*檳榔*)	Tannins; Alkaloid; Arecoline; Arecaidine; Catechin; Epicatechin; Myristic acid; Lauric acid; Palmitic acid; Oleic acid; Linoleic acid	Antitumor; expelling parasite; antiviral; antifungal

**Table 5:** Herbs with reducing phlegm and dehumidification functions with strong FASN inhibitory effect. Phlegm in the body can be not only the pathological product of the body but also a risk factor for tumor development. This group of herbs can relieve symptoms caused by the tumor and have antitumor effects.

*化痰* *去濕* Reduce phlegm and dehumidification	Chemical components	Pharmacological action
*Uncaria gambier *Roxb. (*兒茶*)	Catechu-tannic acid; Catechin; Epicatechin; Phlobatannin; Quercetin; Kaempferol; Afzelechin; Aflatoxin	Inhibition of cancer cell growth; elevation of white blood cells

*Pyrrosia lingua* (Thunb) Farw. (*石葦*)	Diploptene; saponins; Anthraquinone; Flavonoids;	Antitumor; hematischesis; diuresis

*Gleditsia sinensis* Lam. (*皂角刺*)	Flavonoids; Phenol; Fustin; Fisetin	Antitumor

*Eriobotrya japonica *(Thunb.) Lindl. (*枇杷葉*)	Nitrilosides; Farnesol; Amygdalin; Citric acid; Oleanolic Acid; Eugeniin; Tannins	Antitumor; reduce phlegm;

*Morus alba *L. (*桑白皮*)	Flavonoids; Mulberrin; Morusin; Palmitic acid; Essential oils	Inhibition of the growth of tumor; diuresis; detumescence

**Table 6:** Herbs with other functions with strong FASN inhibitory effect. This group of herbs is commonly used and seen in the prescription for malignancy treatment depending on the patient's symptoms.

*其他* Others	Chemical components	Pharmacological action
*Rhuem palmatum *L. (*大黃*)	Puerarin; Chrysophaein; Chrysophanol; Rhein; Gallic acid; Tannins; Catechin	Antitumor; inhibition of the growth of tumor

*Melaphis chinensis *(Bell.) (*五倍子*)	Gallotannin; gallic acid	Antitumor; hematischesis; detoxification

*Artemisia argyi *Levl. et Vant. (*艾葉*)	Essential oils; oil of eucalyptus; Camphor	Antitumor; enhance immune function; bacteriostasis; virus inhibition

*Tetradium ruticarpum* (*吳茱萸*)	Evodiamine; Rutaecarpine; Limonin; Synephrine	Antitumor; bacteriostat; analgesia

*Ramulus Cinnamomi* (*桂枝*)	Cinnamaldehyde; Benzylbenzoate; Cinnamylacetate; Calamenene; Coumarin	Antitumor; antivirus; antibiosis; relieve fever

*Rhizoma Alpiniae Officinarum* (*高良姜*)	Curcumin; Dihydrocurcumin; Galangin; Quercetin; Kaempferol; Kaempferide; Quercetin; Essential oils	Antitumor; antibiosis

**Table 7:** TCM prescriptions with potential FASN inhibitory effect. Among these six commonly used antitumor prescriptions, all of them contain herbs with FASN inhibitory effect. Although studies have shown the antitumor effect of these prescriptions, a lack of studies have been carried out investigating their pharmacological actions and the underlying mechanisms.

Number	Name of the Prescription	Composition	Effect
1	Chaihu Shugan Powder (*柴胡疏肝散*)	Pericarpium Citri reticulatae (*陳皮*); Root of *Chinese thorowax* (*柴胡*); *Rhizoma Ligustici* (*川芎*); Fructus Aurantii (*枳殼*); **The root of *Paeonia lactiflora* Pall**. (*芍藥*); *Radix glycyrrhizae* Preparata (*炙甘草*); *Rhizoma cyperi* (*香附*)	1. Smoothing Liver and Regulating Qi 2. Invigorating Blood Circulation and Alleviate Pain

2	Taoren Chengqi Decoction (*桃核承氣湯*)	*Semen persicae* (*桃仁*); *Radix glycyrrhiza* (*甘草*); Mirabilite (*芒硝*); ***Rhuen palmatum* L**. (*大黃*); ***Ramulus Cinnamomi*** (*桂枝*)	Promoting Blood Circulation for Removing Blood Stasis

3	Fuyuan Huoxue Decoction (*復元活血湯*)	Root of Chinese Thorowax (*柴胡*); Radix Trichosanthis (*瓜蔞根*); Chinese angelica (*當歸*); *Flos carthami* (*紅花*); Radix Glycyrrhizae (*甘草*); Squama manis (*穿山甲*); ***Rhuen palmatum* L**. (*大黄*); Semen Persicae (*桃仁*)	Promoting Blood Circulation for Removing Blood Stasis

4	Xuefu Zhuyu Decoction (*血府逐瘀湯*)	*Chinese angelica* (*當歸*); *Rehmannia glutinosa* Libosch (*生地*); *Semen persicae* (*桃仁*); *Flos carthami* (*紅花*); *Fructus Aurantii* (*枳殼*); ***Paeonia vaitchii* Lynch** (*赤芍*); Root of *Chinese thorowax* (*柴胡*); *Radix glycyrrhizae* (*甘草*); *Platycodon grandiforus* (*桔梗*); *Rhizoma ligustici* Chuanxiong (*川芎*); Radix *Achyranthis bidentatae* (*牛膝*)	1. Promoting Blood Circulation for Removing Blood Stasis 2. Promoting the Qi Circulation and Stopping Pain

5	Wenjing Decoction (*溫經湯*)	***Tetradium ruticarpum*** (*吳茱萸*); *Chinese angelica* (*當歸*); *Rhizoma Ligustici* Chuanxiong (*川芎*) The root of ***Paeonia lactiflora* Pall**. (*芍藥*); *Panax ginseng* C. A. Mey. (*人參*); ***Ramulus Cinnamomi*** (*桂枝*); Colla Corii Asini (*阿膠*) ***Paeonia suffruticosa* Andr**. (*牡丹皮*); *Zingiber officinale* (*生薑*); *Radix glycyrrhizae* (*甘草*); *Pinellia ternata* (*半夏*); *Radix ophiopogonis* (*麥門冬*)	1. Promoting Blood Circulation for Removing Blood Stasis 2. Warming Channel for Dispelling Cold

6	Xiaoyao Powder (*逍遙散*)	Root of *Chinese thorowax* (*柴胡*); *Chinese angelica* (*當歸*); **The root of *Paeonia lactiflora* Pall**. (*白芍*); *Atractylodes macrocephala* (*白術*); *Wolfiporia cocos* (*茯苓*); Rhizoma Zingiberis Recens (*生薑*); *Mentha haplocalyx* (*薄荷*); *Radix glycyrrhizae* Preparat (*炙甘草*)	1. Smoothing Liver and Regulating Qi 2. Promoting Blood Circulation for Removing Blood Stasis

In Composition, Herbal Medicines with the screening of FASN inhibitory effect are bolded.

## Abbreviations

FASN: Fatty acid synthase
TCM: Traditional chinese medicine
CAM: Complementary and alternative medicine
BMI: Body mass index
EGCG: Epigallocatechin gallate
EGFR: Epidermal growth factor receptor
SREBP1C: Sterol regulatory element-binding protein 1c.
